# Identifying potential genetic biomarkers for sperm dysfunction through whole-genome sequencing

**DOI:** 10.1038/s41598-025-23897-w

**Published:** 2025-10-20

**Authors:** Muhammad Riaz Khan, Aftab Ali Shah, Mohammad A. Al Smadi, Nicole Ludwig, Ulrike Fischer, Hashim Abdul-Khaliq, Eckart Meese, Masood Abu-Halima

**Affiliations:** 1 Department of Biotechnology, Faculty of Biological Sciences, University of Malakand, Chakdara, Khyber Pakhtunkhwa Pakistan; 2 Reproductive Endocrinology and IVF Unit, King Hussein Medical Centre, Amman, Jordan; 3 Institute of Human Genetics, Saarland University, 66421 Homburg, Germany; 4 Department of Paediatric Cardiology, Saarland University Hospital, Homburg, Germany

**Keywords:** Male infertility, Whole-genome sequencing (WGS), Pathogenic variants, Genetic biomarkers, Sperm dysfunction, Diseases, Genetics, Medical research, Molecular biology

## Abstract

Infertility affects approximately 15% of couples globally, with male factors contributing to nearly 50% of cases. However, the genetic basis of male infertility, particularly idiopathic forms, remains poorly understood. In this study, we performed whole-genome sequencing (WGS) on eight normozoospermic men and nine men with oligozoospermia, asthenozoospermia, or both, followed by Sanger sequencing for validation. Comparative analysis revealed a higher burden of genomic variants in the sperm dysfunction infertility group (SDIG) than in the normozoospermic group (NG). Several nonsynonymous missense variants were exclusively identified in SDIG, including *DNAJB13* (p.Ile159Asn), *MNS1* (p.Asp217Asn), *DNAH6* (p.Ser2210Leu), *HYDIN* (p.Gly901Ala, p.Arg568Trp), *DNAH7* (p.Arg1486His, p.Gly171Arg, p.Ser2368Phe), *DNAH17* (p.Ala3135Val), and *CATSPER1* (p.Arg558Trp). These variants are predicted to affect protein structure, stability, or interactions, and were classified as variants of uncertain significance. Moreover, several variants were classified as likely pathogenic: a frameshift mutation in *DNAH2* (p.Lys1414ArgfsTer29) likely resulting in a truncated protein, a missense mutation in *CFAP61* (p.Arg568Trp) predicted to impair protein function, and two nonsense mutations in *FSIP2* (p.Gln5809Ter and p.Cys8Ter) introducing premature stop codons. These alterations implicate key components of sperm flagellar function and motility. Our findings reveal novel and potentially deleterious genetic variants associated with male infertility, offering new insights into its molecular underpinnings and informing future diagnostic and therapeutic approaches.

**Supplementary Information:**

The online version contains supplementary material available at 10.1038/s41598-025-23897-w.

## Introduction

Infertility is a widespread global concern, affecting approximately 15% of couples during their reproductive years, equating to about one in every six couples^1,2^. Male factors contribute significantly to this issue, accounting for roughly 30% of infertility cases^1,2^. The World Health Organization defines male infertility as the inability to achieve pregnancy despite regular, unprotected intercourse over a period of at least 12 months. Furthermore, 30% to 45% of male infertility cases involving unexplained semen abnormalities are classified as idiopathic^2,3^. Recent advancements in genomic research have provided valuable insights into the complex genetic underpinnings of reproductive health. These advances are primarily driven by the integration of cutting-edge techniques, such as proteomics, transcriptomics, and microRNA studies, alongside a thorough analysis of their functions^4–8^. An integrative multi-omics method through proteomics, transcriptomics, and small-RNA profiling constantly showed higher payoff in biomarker discovery in infertility compared to single-layer analyses, by repeatedly converging on pathways and candidates across data types^4–8^. Recent reviews in male infertility showcase how cross-omics concordance allows prioritization of genes/regions to be subjected to deeper genomic interrogation and hence restricts whole-genome sequencing (WGS) to functionally implicated networks and loci. Multi-omics atlases of sperm structure and function (e.g., the flagellar ‘microtubulome’) illustrate how proteome–transcriptome–interactome signals may identify high-confidence candidates and then guide variant prioritization in WGS^9–11^.

Given the complexity of spermatogenesis and the numerous genes involved, it is likely that genetic factors play a significant role in male infertility, contributing to approximately 15% of cases^12,13^. Consequently, unravelling the genetic basis of male infertility is a key focus, with identifying specific genes and mutations being crucial for developing targeted diagnostic tools and personalized treatment strategies. Research has increasingly focused on the role of genetic mutations in sperm production and quality, establishing genetic testing as a standard diagnostic tool for certain patients^14^. However, these tests identify the underlying cause in only about 20% of cases, leaving the remaining 80% categorized as idiopathic. This highlights the limitations of current genetic testing and the need for more effective diagnostic methods. Despite extensive testing, around 40% of infertility cases related to impaired spermatogenesis remain unexplained, underscoring a significant gap in our understanding and the necessity for more advanced testing techniques^14,15^. To address this gap, research has examined over 2,000 genes involved in spermatogenesis, their roles and developed more precise diagnostic and therapeutic approaches^7,8,16^. Identifying specific genetic mutations could enable personalized treatments, leading to better fertility outcomes. Advances in sequencing technologies, particularly Next-Generation Sequencing (NGS), have shown promise in identifying potential biomarkers for diagnosing spermatogenesis-related issues^17,18^. However, these methods have not yet revolutionized diagnostic practices.

Recent studies have identified several key genes associated with different forms of male infertility. For instance, mutations in the *DNAJB13* gene have been linked to male infertility issues, including teratozoospermia, a condition where sperm exhibit abnormal morphology^19,20^. These mutations have also been associated with Primary Ciliary Dyskinesia (PCD), a genetic disorder that affects ciliary function and can contribute to infertility^21^. In addition, *MNS1*, another gene implicated in male infertility, has been found to harbor mutations associated with severe oligoasthenoteratozoospermia, a condition characterized by low sperm count, poor sperm motility, and abnormal morphology^22,23^. Genes involved in sperm flagella formation also play a critical role in male fertility. Mutations in *DNAH2* lead to abnormalities in sperm flagella, resulting in asthenoteratozoospermia, which is characterized by poor sperm motility and abnormal flagella structure^24,25^. Similarly, mutations in *DNAH6*, a gene from the same family, have been linked to male infertility and asthenoteratozoospermia, particularly in certain populations, such as the Indian population, where *DNAH6* and *ATPase6* mutations have a significant impact on fertility^26^. Moreover, genes such as *DNAH7*,* DNAH8*,* DNAH9*, and *FSIP2* have been associated with primary infertility, and mutations in *DNAH17* and *CFAP61* contribute to defects in sperm morphology, particularly in the sperm flagella^17,27,28^. Recent studies have also uncovered the role of *FSIP* in sperm acrosome development, where mutations in this gene may lead to globozoospermia, a condition characterized by round-shaped sperm that are unable to fertilize eggs^29^. Additionally, reduced expression of *CatSper1* and *CatSper3*, genes responsible for sperm motility, has been observed in men with asthenoteratozoospermia, leading to poor sperm quality and fertility challenges^30^.

In terms of investigating spermatogenesis and male infertility through NGS, both sperm-derived and blood-derived DNA offer distinct advantages and challenges. DNA isolated from sperm is directly linked to the abnormalities observed in sperm or testicular samples, providing valuable insights into genetic variations, mutations, and epigenetic modifications in germ cells^31,32^. However, the collection process can be difficult, and the samples may exhibit increased variability due to the presence of contaminating cells, such as those from the seminiferous epithelium, somatic cells, or leukocytes^33^. In this study, we aim to identify clinically relevant pathogenic, likely pathogenic, and uncertain significant variants in sperm samples from men with infertility or subfertility. Whole genome sequencing (WGS) was performed on sperm cells from eight men with normozoospermia (normal sperm parameters) and nine men who presented with reduced sperm count, impaired sperm motility, abnormal sperm morphology, or a combination of these infertility-related factors. The primary goal of our research is to uncover genetic variants in the sperm samples of infertile men, including the discovery of novel variants. By identifying these genetic factors, this approach will enhance our understanding of the underlying causes of male infertility and contribute to the development of improved diagnostic tools.

## Materials and methods

### Collection and purification of samples

The samples used in this study were collected from male partners of couples undergoing infertility treatment. All male participants had been experiencing infertility and were unable to conceive after at least 12 months of trying. Sperm samples were collected using a standardized procedure to ensure consistency across the cohort, with each participant following the same collection and handling protocol. The study was conducted after obtaining informed and written consent from all participants and was approved by the Institutional Review Board of the Saarland Medical Association, Saarland, Germany, adhering to ethical guidelines and the Declaration of Helsinki. Two main groups were included: the Normozoospermic Group (NG), consisting of eight men with normal spermiogram results, and the Sperm Dysfunction Infertility Group (SDIG), comprising nine men with conditions such as reduced sperm count (oligozoospermia), diminished sperm motility (asthenozoospermia), abnormal sperm morphology (teratozoospermia), or a combination of these conditions (oligoasthenoteratozoospermia). None of the men in the NG group exhibited teratozoospermia, and all demonstrated normal sperm morphology, with at least 4% of the sperm exhibiting normal morphology. Semen samples were analyzed following the World Health Organization (WHO) 2010 guidelines, incorporating various parameters to assess the normality and subnormality of spermiograms in both groups. To purify the samples and remove somatic cells and debris, 45%-90% PureSperm gradients were used, and the samples were centrifuged at 500 g for 20 min. The pellet was washed twice with Ham-F10 medium containing serum albumin and antibiotics, then overlaid with more medium. Samples were incubated at 37 °C, and after 45 min, the supernatant was separated from the pellet, as previously described^5^. WGS was performed on the sperm samples, and variants were validated using Sanger sequencing.

### Isolation of DNA for subsequent whole-genome sequencing

The extraction of genomic DNA from sperm was conducted using the QIAamp^®^ DNA Mini Kit (Qiagen). The samples were centrifuged at approximately 500 x g for 15 min, with this process repeated five times to ensure comprehensive washing and concentration of the sperm. The washed sperm were then used for DNA isolation, following the manufacturer’s protocol with slight modifications. These adjustments improved the efficiency of DNA release and resulted in higher yields with better purity and integrity, making the material more suitable for downstream WGS analysis. Briefly, 100 µl of sperm eluted in Dulbecco’s Phosphate Buffered Saline (DPBS) was combined with 100 µl of Buffer X2 [20 mM Tris·Cl (pH 8.0), 20 mM EDTA, 200 mM NaCl, 80 mM DTT (freshly added), 4% SDS, and 250 µg/ml Proteinase K (freshly added) (Sigma-Aldrich). The resulting 200 µl mixture underwent incubation at 55 °C for 1 h, being periodically inverted every 15 min. Following incubation, 200 µl of Buffer AL from the QIAamp^®^ DNA Mini Kit and 200 µl of ethanol were added to each sample. The samples were thoroughly mixed by vortexing and then settled by brief centrifugation. Subsequently, the procedure was completed according to the Qiagen recommendations, starting from steps 5 to 8 of the Tissue Protocol in the QIAamp DNA Mini Kit handbook. DNA was eluted twice, with each elution using 25 µl of Buffer AE (Qiagen) and an incubation period of 3 min at room temperature. Finally, the quantity and quality of DNA were assessed using NanoDrop™ 2000c Spectrophotometers and Qubit™ 4 Fluorometer (Thermo Fisher Scientific), and the integrity of the DNA was evaluated using the Agilent 2100 Bioanalyzer (Agilent Technologies) as well as conventional 1% agarose gel electrophoresis.

### DNA library Preparation and sequencing

Following the assessment of DNA quantity and integrity, 100 ng of DNA from each sample was employed according to the Library Construction Protocol from the MGIEasy FS DNA Library Prep Manual Version: B4 (MGI Technologies). In brief, genomic DNA underwent fragmentation with a mixture containing Frag Buffer II and Frag Enzyme II on ice, following the prescribed MGI program on the thermocycler. Post-fragmentation, the resulting products were purified using DNA Clean Beads (MGI Technologies) and quantified via the Qubit^®^ dsDNA HS Assay Kit on the Qubit 4™ Fluorometer (Thermo Fisher Scientific). For the subsequent step, 100 ng of fragmentation products were subjected to end repair and A-tailing, employing ERAT Buffer and ERAT Enzyme Mix on ice, adhering to the recommended MGI program on the thermocycler. Subsequently, MGIEasy DNA Adapters, Ligation Buffer, and DNA Ligase were added to the mixture, which was then loaded onto the thermal cycler and incubated at 23^°^C for 30 min. A further purification step using DNA Clean Beads was conducted as per MGI recommendations. Following this, a PCR amplification mixture consisting of PCR Enzyme Mix and PCR Primer Mix was prepared on ice. Using a cycling program of 7 cycles, the resulting products were once again purified using DNA Clean Beads. Quantification of the purified PCR products was performed using the Qubit^®^ dsDNA HS Assay Kit, and the fragment size distribution was assessed using a High Sensitivity DNA kit (Agilent Technologies). Subsequent steps involved desaturation of PCR products at 95^°^C for 3 min, followed by single-strand circularization using a mixture of Splint Buffer and DNA Rapid Ligase on ice, with subsequent incubation at 37^°^C for 30 min. Following circularization, enzymatic digestion of single-stranded products was carried out using a mixture of Digestion Buffer and Digestion Enzyme at 37^°^C for 30 min. The resulting digested products were purified using the DNA Clean Beads protocol, as detailed in the MGI operating manual. The final product was subjected to sequencing on the MGI BGISEQ-G400 (MGI Technologies), following the manufacturer’s instructions.

### DNA sequencing data analysis

#### Processing of DNA sequencing data

In the DNA sequencing data processing, various statistical analysis procedures were applied, commencing with unique barcoding and multiplexing. Each sample was uniquely barcoded to facilitate multiplexing during sequencing, allowing for the subsequent separation of sequence raw data into individual lanes based on these barcodes. Subsequently, the raw data underwent demultiplexing, a crucial process that separated mixed sequences based on their distinct barcodes, ensuring alignment with their respective samples, and streamlining downstream analyses.

#### Data consolidation and quality checks

Following multiplexing and demultiplexing, raw data from different lanes sharing the same barcode were combined for mutation analysis. This consolidation generated single first and second-pair files for each unique barcode, providing a streamlined dataset for further analysis. Before proceeding with subsequent steps, a raw read quality check was performed using FastQC (Version 0.11.9; https://www.bioinformatics.babraham.ac.uk/projects/fastqc/) on the combined FastQ files. To enhance data quality, trimming was conducted using the Cutadapt algorithm^34^ (Version 4.0; https://cutadapt.readthedocs.io/). The quality cutoff for trimming (-q 30,30) was set to enforce a minimum quality score of 30 for both the 5’ and 3’ ends of the sequencing reads. Additionally, a minimum length for read retention after adapter removal was specified with (-m 20), excluding reads shorter than 20 bases following trimming. A second round of quality checks using FastQC post-trimming was carried out to ensure the overall improvement in sequence data quality.

#### Comprehensive DNA sequencing pipeline

We implemented a comprehensive DNA sequencing pipeline to systematically investigate genetic information. The process was initiated with the alignment of raw sequencing reads to a reference genome (GRCh38), facilitated by the Burrows–Wheeler Aligner (BWA-MEM, Version 0.7.17; http://bio-bwa.sourceforge.net/) algorithm^35^. To optimize sequence searches, a reference genome index was generated before mapping through the execution of the ‘bwa index’ command. Subsequently, the actual mapping of reads was conducted using the ‘bwa mem’ command. The resulting Sequence Alignment/Map (SAM) file was transformed into a sorted Binary Alignment/Map (BAM) file using the SAMtools suite (Version 1.16.1; http://www.htslib.org/) with the ‘samtools view’ and ‘samtools sort’ commands^36^.

#### Quality assessment and mutation analysis

To assess the quality of the alignment and counts data, the QualiMap algorithm (Version 2.3; http://qualimap.conesalab.org/) was utilized^37^. Identification and marking of duplicate sequences were performed using Picard tools (Picard Toolkit, Version 2.27.4; Broad Institute; https://broadinstitute.github.io/picard/), followed by the augmentation of BAM file headers through the ‘AddOrReplaceReadGroups’ program from Picard tools. The marked and header-enhanced BAM file was then subjected to indexing using the ‘samtools index’ command^36^.

#### Mutation calling and recalibration

For mutation calling, the Genome Analysis Toolkit (GATK, Version 4.3.0.0; https://gatk.broadinstitute.org/) was employed^38^. This necessitated the prior generation of genome dictionary files with the ‘Picard-tools CreateSequenceDictionary’ command and the indexing of the reference genome using ‘samtools faidx’ ^36^. Next, the HaplotypeCaller function was applied with specified parameters to analyze the input BAM file and generate a variant call format (VCF) file^38^. Subsequently, the identified mutations underwent stringent filtering based on GATK standard criteria for both single-nucleotide polymorphisms (SNPs) and insertions/deletions (indels). Crucially, within GATK, the term “indel” encompasses both insertions and deletions. SNPs were subjected to filters targeting quality, strand bias, mapping quality, strand odds ratio, and various rank sum tests. Indels were similarly filtered with specific criteria. Recalibration of base quality scores was conducted using known sites from predicted mutations and SNP database mutations. The recalibrated data were then applied to the original BAM file. The final variation calling involved using the recalibrated BAM file with the HaplotypeCaller function. Further refinement of the variations was performed using SnpSift (Version 5.1; part of the SnpEff suite; http://snpeff.sourceforge.net/) ^39^, applying filters for quality (QUAL > = 30), mapping quality (MQ = 30), and depth (DP > = 10).

#### Variant filtering and annotation

In the variant annotation process, we utilized the VarSome Clinical tool (Version 13.1.1; https://clinical.varsome.com/), which is specifically designed for the analysis, annotation, and prioritization of genomic variants^40^. This tool was used to filter the variants in each VCF file for each tested sperm sample. The initial data filtration was performed using seven stringent criteria. First, variants were filtered based on pathogenicity, categorized as Pathogenic (P), Likely Pathogenic (LP), Uncertain Significance (VUS), and their subclasses: VUS with Pathogenic Potential (VUS|P), VUS with Likely Pathogenic Potential (VUS|LP), VUS with Benign Potential (VUS|B), while variants classified as Likely Benign or Benign were excluded from further consideration. Second, variants were classified according to the Online Mendelian Inheritance in Man (OMIM) system, considering autosomal recessive, autosomal dominant, X-linked, Y-linked, mitochondrial inheritance, and those classified as homozygous with the variant, homozygous with the reference, heterozygous, heterozygous phased, or mitochondrial. Third, variants in the VCF files were further examined for their functional impact, including coding effects such as frameshift, nonsynonymous missense, nonsense, stoploss, exon deletion, in-frame, start loss, and splice junction loss, which are likely to adversely affect protein function. Fourth, the Combined Annotation-Dependent Depletion (CADD) score was applied to assess the potential impact of genetic variants on human health (CADD, https://cadd.gs.washington.edu/)^41^, with variants having a CADD score equal to or greater than 20 considered for further analysis. Fifth, since the DNA was extracted from purified sperm samples of men with infertility, variants were cross-referenced against 645 sperm-specific genes with elevated expression in the sperm compared to other tissues, as indicated in the Human Protein Atlas (https://www.proteinatlas.org/)^42^. Genes were matched with testis-specific proteins from the Human Protein Atlas, and only those with alignment were selected for further investigation. Sixth, variants were filtered for their potential relevance to infertility-related pathways and conditions. These variants were then further annotated according to the American College of Medical Genetics and Genomics (ACMG) guidelines^43^. Seventh and finally, in silico predictors were used to assess the potential impact of the variants, all integrated into the VarSome Clinical tool.

### Validation of genetic variants using Sanger sequencing

Sanger sequencing was performed to confirm and validate selected variants based on the availability of remaining DNA following initial clinical and WGS analyses. Sanger sequencing was performed using DNA isolated from each sample. Initial PCR amplification was carried out using primers listed in Supplemental Table S1, with MyTaq™ HS Red Mix in a 50 µL reaction volume containing 2 µL DNA (10 ng/µL). PCR products were analyzed via 1.5% agarose gel electrophoresis to verify the expected band sizes based on Supplemental Table S1. Following confirmation, DNA bands of the correct size were excised from the gel after staining with GelStar™ and visualization under blue light. The excised gel fragments were purified using the NucleoSpin^®^ Gel and PCR Clean-up kit, with minor modifications to the standard protocol. DNA was eluted in 30 µL Elution Buffer and quantified using a NanoDrop spectrophotometer. Purified products were then submitted to Eurofins Genomics for Sanger sequencing. Sequences were aligned using BLAST (BLAST + v2.17.0; https://blast.ncbi.nlm.nih.gov/Blast.cgi*)* to confirm unique genomic positions, and variants were manually verified through chromatogram inspection.

## Results

### Evaluation of the demographic, hormonal, and clinical characteristics

Sperm parameters, IVF outcomes, and hormone profiles of female partners were compared between two groups of male participants: the SDIG group (*n* = 9) and the NG group (*n* = 8). The NG group consistently exhibited superior semen quality in terms of sperm concentration, motility, and morphology compared to the SDIG group. The average progressive motility was 51.1% in the NG group versus 34.4% in the SDIG group, while morphology and vitality scores also favored the NG group (average morphology: 4.25% in NG vs. 1.94% in SDIG; average vitality: 69% in NG vs. 55.6% in SDIG).

In the SDIG group, lower sperm quality metrics were correlated with poorer IVF outcomes. Several samples, such as IDs 290, 220, 271, 285, 287, and 282, had negative pregnancy outcomes, although the hormonal profiles of their female partners (i.e., FSH, LH, PRL) were within normal physiological levels. Importantly, sample 287 had a normal sperm count but low progressive motility (24%) and did not achieve embryo cleavage, in favor of the presence of genetic mutations involving sperm function. Conversely, three samples in the SDIG group, 279, 201, and 203, had positive β-hCG outcomes, despite only moderate sperm parameters, suggesting that while sperm quality is influential, it is not the sole predictor of IVF success.

In the NG group, all participants underwent intrauterine insemination (IUI). Despite overall strong semen profiles, four samples (IDs 275, 280, 281, and 286) resulted in negative pregnancy outcomes, reinforcing the idea that factors beyond standard sperm parameters, such as genetic, epigenetic, or uterine receptivity factors, may impact success rates. However, samples 175 and 274 in the NG group did result in positive pregnancies, aligning with their superior sperm metrics. These observations suggest that while sperm parameters are essential indicators of fertility potential, they must be interpreted alongside other clinical and molecular factors for accurate prognostication in assisted reproduction outcomes.

### Comparative genomic analysis of groups

Our analysis uncovered distinct patterns between the Normozoospermic Group (NG) and the Sperm Dysfunction Infertility Group (SDIG) across several genomic metrics. Supplemental Table S2 provides a detailed comparison between the two groups, “NG, n = 8” and “SDIG, n = 9,” incorporating key metrics such as the average count of mapped reads, percentage of reads successfully mapped, GC content percentage, coverage, and mapping quality. In the SDIG group, the average count of mapped reads was approximately 359.7 million, ranging from about 258 million to 505 million. The percentage of successfully mapped reads averaged 99.5%. The GC content percentage was similar to the NG group, averaging 39.8%. The coverage varied, ranging from 11.68x to 22.62x, with an average of 15.90x. The mapping quality was consistently high, with an average of 31.94.

In contrast, the NG group had a slightly lower average count of mapped reads, around 349.7 million, yet maintained a high percentage of successfully mapped reads, averaging 99.87%. The average GC content percentage was comparable to the SDIG group, at approximately 39.59%. However, the average coverage was slightly lower at 15.47x. The mapping quality remained consistently high, with an average of 31.92.

### Analysis of genetic consequences in SDIG and NG

Comparing the genetic consequences between SDIG and NG revealed notable differences across various genomic parameters. Supplemental Table S3 provides a comprehensive comparison between the two groups, “SDIG, n = 9” and “NG, n = 8,” covering all genetic consequences, including those related to coding regions. For instance, the NG group exhibited a slightly higher count in Transcript Ablation and Incomplete Terminal Codon Variants, indicating higher variance for these consequences. A substantial difference was observed in the counts of the 5’UTR variant, with the NG group showing a significantly higher count. Although NG also showed notable concentrations of various coding consequences (e.g., stop gained, frameshift variant), the SDIG group had higher counts in most categories, suggesting a greater prevalence of these consequences. These disparities could potentially be linked to the differences in fertility between the two groups (Table 1).

**Table 1:** Clinical characteristics and standard semen parameters.

Sample ID	Age (years)	Volume (ml)	pH	Sperm count (million/ml)	Progressive motility (PR, %)	Non-progressive motility (NP, %)	Immotile (IM, %)	Morphology (%)	Vitality (%)	IVF outcome parameters	Female partner hormonal parameters			
										Embryo quality grade	β-hCG	FSH (mIU/ml)	LH (mIU/ml)	PRL (ng/ml)
**Male partner sperm parameters (SDIG** ***n= 9***)														
290	46	6.2	7.7	1.2	52	7	41	2	67	1G2/1G3	Negative	11	17	22.4
220	40	2.6	7.5	3.7	48	6	46	2.5	55	1G1/2G2	Negative	5.5	7.8	7.0
279	37	3.2	8.0	4.2	22	9	69	2.5	44	1G1/2G2	Positive	9.7	12	15.2
271	45	2.9	7.8	7.6	26	12	62	2	44	2G2/1G3	Negative	8.4	6.5	9.8
201	35	3.2	8.0	7.8	33	12	55	2	55	2G1/1G2	Positive	6.6	9.2	8.0
285	39	6.2	7.8	10	52	6	42	2	73	1G2/1G3	Negative	11	9	11.5
203	34	4.9	8.5	11	33	14	53	1.5	52	1G1/2G2	Positive	5.2	7.1	8.8
287	48	4.0	7.5	12	24	4	72	2	55	No cleavage	Negative	9.1	12	7.8
282	30	2.3	8.3	18.5	20	9	71	2	55	2G2/1G3	Negative	4.2	11	21.4
Mean ± SD	**39.33 ± 5.70**	**3.94 ± 1.41**	**7.90 ± 0.32**	**8.44** **± 4.91**	**34.44 ±** **12.24**	**8.78** **± 3.15**	**56.78 ± 11.60**	**2.06 ± 0.28**	**55.56 ± 8.92**			**7.86 ± 2.42**	**10.18 ± 3.07**	**12.43 ± 5.57**
**Male partner sperm parameters (NG *n = 8***)														
270	30	3.3	8	54	58	5	37	4	69	IUI	Negative	4.3	9.1	8
175	34	7.7	7.8	58	61	11	28	4	77	IUI	Positive	7.8	5.4	7
251	34	2.0	7.7	62.3	32	6	62	5	74	IUI	Negative	8	3.8	14
280	33	2.2	8.0	69	58	16	26	4	82	IUI	Negative	7	9	20.1
275	42	4.9	7.7	71	46	8	46	4	55	IUI	Negative	12	15	21.5
286	39	1.9	7.7	82	41	4	55	5	72	IUI	Negative	9.8	12	10.3
281	35	1.8	8.0	95	47	5	48	4	60	IUI	Negative	7.8	11	21
274	29	6.4	8.0	96	46	8	46	4	63	IUI	Positive	7.8	5.4	9.5
Mean ± SD	**34.50 ± 4.03**	**3.78 ± 2.15**	**7.86 ± 0.14**	**73.41 ± 15.05**	**48.62** **± 9.22**	**7.88** **± 3.72**	**43.50 ± 11.70**	**4.25** **± 0.43**	**69.00 ± 8.51**			**8.06 ± 2.06**	**8.84 ± 3.57**	**13.93 ± 5.72**
p-value	**0.076**	**0.862**	**0.770**	**< 0.001**	**0.022**	**0.623**	**0.044**	**< 0.001**	**0.009**			**0.860**	**0.452**	**0.617**

Values are expressed as mean ± standard deviation (SD) unless otherwise indicated. Comparisons between the SDIG (Sperm Dysfunction Infertility Group, *n* = 9) and NG (Normozoospermic Group, *n* = 8) were performed using the Mann–Whitney U test for continuous variables. A p-value < 0.05 was considered statistically significant.

SDIG, Sperm Dysfunction Infertility Group; NG, Normozoospermic Group; β-hCG, Beta human chorionic gonadotropin; FSH, Follicle-stimulating hormone; LH, Luteinizing hormone; PRL, Prolactin; G1, Embryo Quality Grade 1; G2, Embryo Quality Grade 2; G3, Embryo Quality Grade 3; IUI, Intrauterine Insemination.

### Interpretation of deleterious variants

WGS was conducted on 17 samples (9 SDIG and 8 NG), categorized based on semen analysis results. The data were analyzed using the GATK pipeline. Following the software guidelines, the testing pipeline was executed sequentially, utilizing custom shell scripts to generate the VCF files. Variant analysis, annotation, and prioritization were then carried out using the VarSome Clinical tool, and the list of variants identified are listed in Table 2. It was interesting to note that all the female partners of patients No.285, 220 and 287, where a missense SNV (NM_001370.2 c.6629 C > T (p.Ser2210Leu) at position chr2:84677021 C > T, NM_018365.4 c.649G > A (p.Asp217Asn) at chr15:56444481 C > T and NM_015585.4 c.1702 C > T (p.Arg568Trp) at chr20:20196681 C > T) was reported in *DNAH6*,* MNS1* and *CFAP61* respectively were β-hCG negative and ultimately led to negative pregnancy.

**Table 2:** Candidate diagnostic variants identified in each sample.

Sample ID	Gene	NT genomic change (GRCh38)	Consequence	Variant	Zygosity	CADD / PolyPhen2 / SIFT	Allele frequency	ACMG
290	*DNAJB13*	chr11:73965019 T > A	Nonsynonymous SNV	ENST00000339764.6 c.476T > A (p.Ile159Asn)	Het	26.6/D /P		VUS
220	*MNS1*	chr15:56444481 C > T	Missense SNV	NM_018365.4 c.649G > A (p.Asp217Asn)	Het	27.1/D/P		VUS
271	*DNAH2*	chr17:7770811 delA	Deletion	ENST00000572933.6 c.4241del (p.Lys1414ArgfsTer29)	Het	NA		Likely Pathogenic
285	*DNAH6*	chr2:84677021 C > T	Missense SNV	NM_001370.2 c.6629 C > T (p.Ser2210Leu)	Het	23.9/D	0.000	VUS
287	*CFAP61*	chr20:20196681 C > T	Missense SNV	NM_015585.4 c.1702 C > T (p.Arg568Trp)	Homo	27.28/D/ P	0.001151	Likely Pathogenic
287	*HYDIN*	chr16:71031745 C > G	Missense SNV	NM_001270974.2 c.2702G > C (p.Gly901Ala)	Het	22.2/D		VUS
282	*HYDIN*	chr16:71129811 delATC	In-frame Deletion	NM_001270974.2 c.1702 C > T (p.Arg568Trp)	Het	NA		VUS

Sample ID	Gene	NT genomic change (GRCh38)	Consequence	Variant		CADD / PolyPhen2 / SIFT		ACMG
275	*DNAH7*	chr2:195900373 C > T	Missense SNV	NM_018897.3 c.4457G > A (p.Arg1486His)	Het	28/D/P	0.000	VUS
275	*DNAH7*	chr2:196026916 C > T	Missense SNV	NM_018897.3 c.511G > A (p.Gly171Arg)	Het	25.6/D/P		VUS
275	*DNAH17*	chr17:78460193 G > A	Missense SNV	ENST00000389840.7 c.9404 C > T (p.Ala3135Val)	Het	26/P	0.000	VUS
280	*FSIP2*	chr2:185806731 C > T	Nonsense SNV	NM_173651.4 c.17,425 C > T (p.Gln5809Ter)	Het	32/D/P		Likely Pathogenic
280	*FSIP2*	chr2:185738918 C > A	Nonsense SNV	NM_173651.4 c.24 C > A (p.Cys8Ter)	Het	35/P	0.000	Likely Pathogenic
280	*DNAH8*	chr6:38842875 C > A	Missense SNV	NM_001206927.2 c.4817 C > A (p.Pro1606Gln)	Het	22/D	0.000	VUS
281	*DNAH7*	chr2:195864552 G > A	Missense SNV	ENST00000312428.11 c.7103 C > T (p.Ser2368Phe)	Het	26/P	0.000	VUS
281	*CATSPER1*	chr11:66021515 G > A	Missense SNV	NM_053054.4 c.1672 C > T (p.Arg558Trp)	Het	26.6/D/P	0.0001644	VUS

PD, Probably Damaging; D, Damaging; DLC, Damaging (Low Confidence); P, Pathogenic; SNVs, Single Nucleotide Variants; NT, Nucleotide; SNV, Single Nucleotide Variant; Het, Heterozygous; Homo, Homozygous; CADD, Combined Annotation Dependent Depletion; PolyPhen2, Polymorphism Phenotyping v2; SIFT, Sorting Intolerant From Tolerant; ACMG, American College of Medical Genetics and Genomics; VUS, Variant of Uncertain Significance.

#### Nonsynonymous SNV in *DNAJB13*

A single nucleotide missense variant (SNV) was identified in the *DNAJ* heat shock protein family (*Hsp40*) member B13 (*DNAJB13*), located on chromosome 11 at position 73,965,019. This mutation is characterized by a thymine to adenine change (T > A) at position c.476T > A, resulting in the replacement of isoleucine with asparagine (I159N, p.Ile159Asn). The variant follows a heterozygous autosomal recessive inheritance pattern. Its clinical significance is classified as a VUS. According to OMIM, this variant is associated with ciliary dyskinesia.

#### Missense SNV in *MNS1*

A missense SNV was detected in the Meiosis-Specific Nuclear Structural 1 (*MNS1*) gene, located on chromosome 15 at position 56,444,481. This mutation involves the substitution of cytosine with thymine (C > T) at position c.649G > A. The variant is heterozygous and is situated within the coding region of the gene. This substitution leads to the replacement of aspartic acid (p.Asp217Asn) with asparagine, and the CADD score predicts this variant to be likely pathogenic.

####  Detection of point mutations in four members of the DNAH gene family

The dynein axonemal heavy chain gene family (*DNAH*) comprises twelve members: *DNAH1*,* DNAH2*,* DNAH3*,* DNAH5*,* DNAH6*,* DNAH7*,* DNAH8*,* DNAH9*,* DNAH10*,* DNAH11*,* DNAH12*, and *DNAH17*. Several point mutations were detected in four of these genes:

- *DNAH2*: An in-frame deletion was identified in *DNAH2*, located on chromosome 17 at position 7,770,811. This frameshift deletion involves the loss of adenine at position c.4241del. The variant follows an autosomal recessive inheritance pattern and is classified as likely pathogenic. The mutation results in a change in the coding frame, causing a structural alteration in the protein at position (p.Lys1414ArgfsTer29), where lysine is replaced by arginine.
- *DNAH6*: A missense SNV was detected in *DNAH6*, located on chromosome 2 at position 84,677,021 (c.6629 C > T) in exon 41. The detected variant follows an autosomal recessive inheritance pattern in the heterozygous state. This mutation causes the substitution of serine by leucine at position (p.Ser2210Leu), leading to a potential alteration in protein function.
- *DNAH7*: A missense variant was found in *DNAH7*, located on chromosome 7. This mutation follows an autosomal recessive inheritance pattern and leads to a substitution of guanine with adenine at position c.511G > A in exon 7. At the protein level, glycine is replaced by arginine at position (p.Gly171Arg).
- *DNAH8*: Similarly, a missense variant was detected in *DNAH8*, located on chromosome 6 at position 38,842,875 (c.4817 C > A). The mode of inheritance is autosomal recessive and heterozygous, with the mutation present on exon 35 (NM_001206927.2). This point mutation results in the replacement of proline with glutamine at the protein level (p.Pro1606Gln).
- *DNAH17*: A missense point mutation was identified in *DNAH17*, located on chromosome 17 at position 78,460,193. The mutation is a substitution of G > A at position c.9404 (C > T) on exon 59. This autosomal recessive heterozygous alteration leads to the replacement of alanine with valine at position (p.Ala3135Val), potentially impacting protein structure and function.

#### A missense SNV in *FSIP2*

A missense variant was detected in the fibrous sheath interacting protein 2 (*FSIP2*) gene, located on chromosome 2. Two mutations were identified in different positions: chr2:185806731 (c.17425 C > T) and chr2:185738918 (c.24 C > A). Both mutations are heterozygous and follow an autosomal recessive inheritance pattern. These mutations are predicted to be likely pathogenic based on the CADD score, SIFT, and PolyPhen-2. These genetic alterations (p.Gln5809Ter), (p.Cys8Ter) in *FSIP2* cause alterations in protein structure.

#### A missense SNV in *HYDIN*

In exon 19 of the axonemal central pair apparatus protein (*HYDIN*) gene (transcript NM_001270974.2), a missense SNV was detected at position chr16:71031745 (c.2702G > C). This mutation is autosomal recessive and heterozygous, and it is classified as a variant of uncertain significance. The substitution of guanine by cytosine leads to structural changes in the protein, resulting in the replacement of glycine by alanine at position p.(Gly901Ala). Additionally, in the same patient, another point mutation was detected in the *CFAP61* gene located on chromosome 20 at position 20,196,681 (c.1702 C > T) on exon 16. This mutation causes the replacement of arginine by tryptophan at position p.(Arg568Trp).

#### Point mutation in *CATSPER1*

A missense point mutation was identified in the cation channel sperm associated 1 (*CATSPER1*) gene, located on chromosome 11. The variant is autosomal recessive and heterozygous, with predictions of being pathogenic based on the CADD score and deleterious according to SIFT. This mutation (G > A) at position chr11:66021515 (c.1672 C > T) disrupts the structural arrangement of the protein, leading to the replacement of arginine with tryptophan at position p.(Arg558Trp).

### Validation of WGS results by Sanger sequencing

Sanger sequencing was conducted to confirm the variants detected by whole-genome sequencing (WGS) in the *DNAH6*,* MNS1*, and *CFAP61* genes. These genes were prioritized for validation on the basis of the quantity of residual DNA material after WGS and the clinical significance of the detected variants. Other variants found in other genes could not be validated by Sanger sequencing because of DNA amount limitations.

**Fig. 1:**
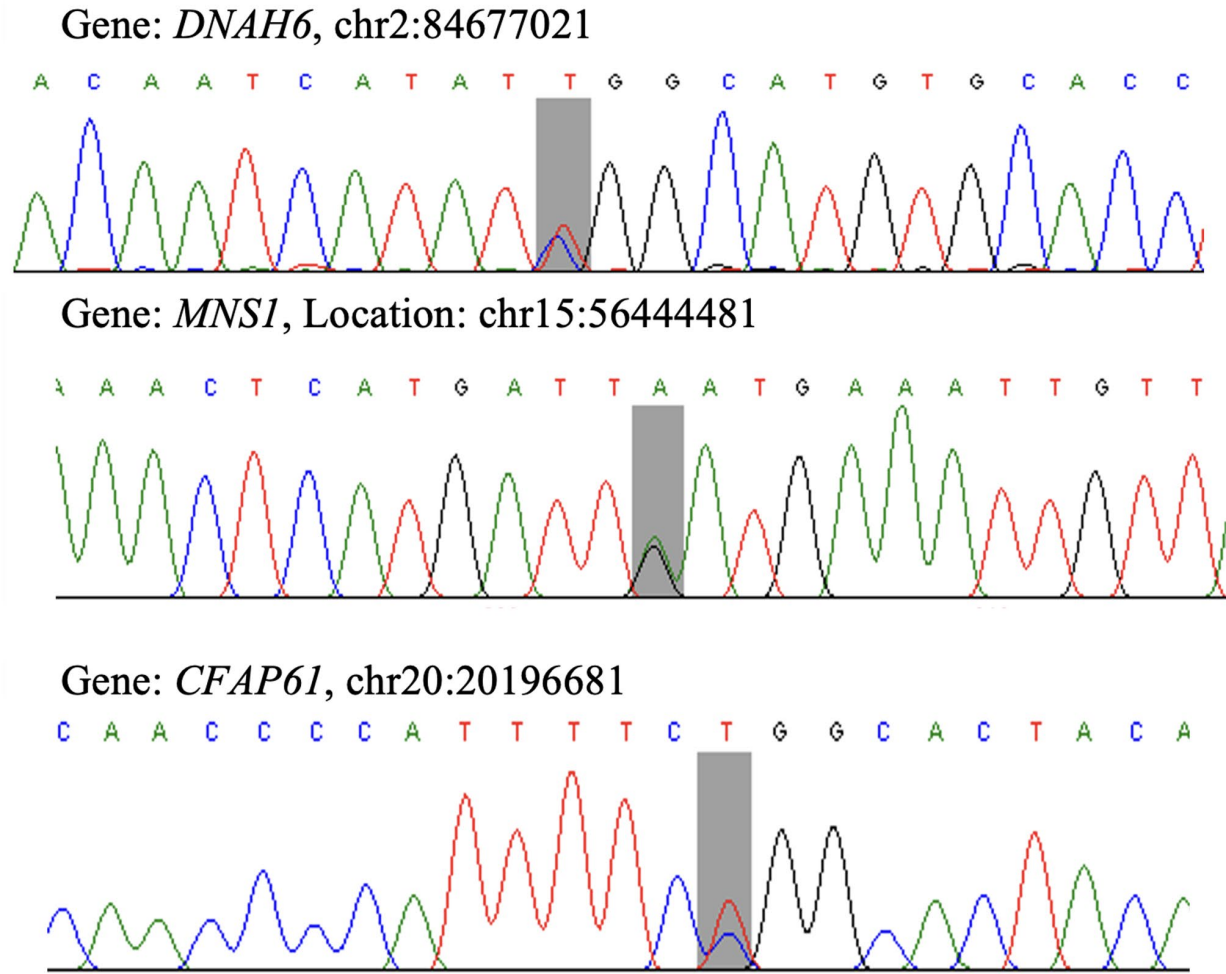
The Sanger sequencing chromatograms validating the variants detected through WGS in *DNAH6*, *MNS1*, and *CFAP61* genes in SDIG, which were absent in NG. The mutation position is highlighted with a grey colour.

Notably, the complementary strand of *MNS1* was reverse-primer sequenced, hence the variant has been described as G > A rather than C > T, as indicated in Fig. 1. The concordance of WGS and Sanger sequencing results is a reflection of the accuracy and reliability of the variant calls and confirmation of the presence of these alterations in genes associated with male infertility. Although only *DNAH6*,* MNS1*, and *CFAP61* have been confirmed, the remaining candidate genes uncovered by whole genome sequencing, *DNAJB13*,* HYDIN*,* DNAH7*,* DNAH17*,* FSIP2*,* DNAH8*, and *CATSPER1*, are also very likely to contain functionally important variants, pending verification.

## Discussion

Genetic elements are essential in the intricate process of spermatogenesis, and disruptions in these genes can lead to male infertility. High-throughput sequencing has paved the way for identifying genes or variants involved in spermatogenesis. However, genetic variants such as indels, sequence alterations, insertions, deletions, and single-nucleotide variants (SNVs) only account for a portion of idiopathic male infertility cases, suggesting that other genetic or epigenetic factors may also contribute^44^. The genetic mechanisms behind most of these variants in male reproductive abnormalities remain largely unknown, emphasizing the need for further research. This research work highlights the importance of genetic alterations in better understanding the molecular mechanism of male infertility.

In this study, WGS was performed on purified sperm samples from two groups: men with normal spermiogram results (normozoospermic men) and men with oligozoospermia, asthenozoospermia, combined with teratozoospermia, or oligoasthenoteratozoospermia. Both groups demonstrated efficient mapping processes with high percentages of successfully mapped reads and comparable GC content. The SDIG group exhibited greater variability in coverage compared to the NG group, with a broader range and higher average. Despite this variability, mapping quality remained consistently high across both groups. Notably, the SDIG group had slightly higher averages in mapped reads and coverage, suggesting potential insights into the biological processes related to male infertility. Factors such as individual differences, experimental conditions, or other unidentified variables may contribute to these variations within each group. The detected variants range from key candidate genes, including members of the DNAH family, *DNAJB13*, *MNS1*, *FSIP2*, *HYDIN*, and *CATSPER1*. It has already been reported that genetic variations in the genes belonging to the DNAH family are known to be associated with cilia-related disorders, such as immotile cilia syndrome^45^. Genetic alteration in *DNAH2* contributes to defects in sperm flagella, leading to abnormal sperm motility^46^. *DNAJB13* is a member of the DNAJ/HSP40 family of proteins, known to play a critical role in regulating axonemal dynein arms and sperm motility^21^. In mature spermatozoa, *DNAJB13* is localized along the entire length of the sperm flagellum, suggesting that it plays an essential role in the formation and function of sperm flagella^47^. In our study, we identified a nonsynonymous variant, c.476T > A; p.Ile159Asn, in *DNAJB13*, which follows an autosomal recessive inheritance pattern, further supporting its involvement in male infertility. *MNS1* is crucial for the assembly of sperm flagella and the motility of cilia^22^. Previous studies have highlighted biallelic *MNS1* variants as a risk factor for male infertility^48^. Loss-of-function (LOF) mutations in *MNS1* have been linked to Oligoasthenoteratozoospermia in a Han Chinese patient, and other studies have shown that LOF mutations also contribute to laterality defects and male infertility^23,49^. In alignment with these findings, we reported a missense SNV mutation (*c.649G > A; p.Asp217Asn*) in *MNS1* that is likely to contribute to sperm dysfunction. *DNAH2* is a member of the DNAH family, and previous studies have shown that mutations in this gene lead to Oligoasthenozoospermia^24^.

Our findings are consistent with these reports, identifying a frameshift deletion (*c.4241delA; p.Lys1414ArgfsTer29*) that disrupts a significant protein structure. This suggests that mutations in *DNAH2* may contribute to both primary ciliary dyskinesia and asthenozoospermia, further reinforcing the gene’s role in sperm flagellar function. Similarly, *DNAH6* is expressed in the testis and localized to the neck region of the spermatozoa. Mutations in this gene have been linked to abnormalities in sperm heads^50^ and have been implicated in conditions such as primary ciliary dyskinesia and non-obstructive azoospermia^51,52^. Our study corroborates these findings, identifying a missense SNV (*p.Ser2210Leu*) in *DNAH6*, which causes a substitution of serine with leucine, leading to alterations in the protein structure. Moreover, using whole-exome sequencing (WES), a study identified homozygous nonsense mutations in *DNAH7* that cause abnormalities in human sperm flagella^53^. Our study also detected a missense variant in *DNAH7*, further supporting its role in male infertility. Previous studies have also reported novel homozygous splicing variants in *DNAH7* that lead to frameshift mutations^54^. Furthermore, *DNAH17* has been shown to follow an autosomal recessive inheritance pattern, with ten novel mutations identified in previous studies^55^. In our study, we detected mutations in *DNAH2*, *DNAH6*, *DNAH7*, *DNAH8*, and *DNAH17*, all of which are crucial for sperm motility and reproductive health in males. Primary ciliary dyskinesia (PCD) is a heterogeneous disorder characterized by mutations in over 50 genes, with most linked variants following an autosomal recessive mode of inheritance 52. According to Knowles et al., nearly 70% of PCD-related genes are screened using targeted gene panels, suggesting that additional causative genes may exist^56^.

Our study identified mutations in genes that are highly relevant for PCD, indicating that these genetic alterations may also play a role in male infertility. We also observed that mutations in *FSIP2* lead to multiple morphological abnormalities in the sperm flagella^57^. Previous studies have shown that missense variants in *FSIP2* result in milder damage to spermatozoa 55. Pathogenic mutations in *FSIP2* have been associated with multiple morphological abnormalities of the flagella (MMAF), and our study aligns with these findings, identifying potential variants (*p.Gln5809Ter* and *p.Cys8Ter*) that disrupt *FSIP2* protein structure in our study subjects. Additionally, we detected a point mutation in *HYDIN* (*c.2702G > C; p.Gly901Ala*), a gene previously associated with loss-of-function variants in male infertility 56. All variants detected in this study, including nonsynonymous SNVs in *DNAJB13*, *MNS1*, *DNAH6*, *DNAH7*, *DNAH8*, *DNAH17*, *FSIP2*, *HYDIN*, *CFAP61*, and *CATSPER1*, as well as an in-frame deletion in *DNAH2*, were heterozygous and followed an autosomal recessive inheritance pattern. These findings offer new insights into the genetic mechanisms underlying male subfertility and provide opportunities for early diagnosis and intervention^58^.

According to data from the Human Protein Atlas and Genotype-Tissue Expression (GTEx), these genes are expressed in various tissues and are particularly relevant in reproductive biology due to their specific expression in the testis, as shown in Supplemental Figure S1 The variants identified in our study not only reveal additional variants that could be associated with male infertility but also validate previously recognized variants within the genomic landscape of male infertility. This research specifically examines purified human sperm samples from both the SDIG and the NG. These findings have important implications for enhancing diagnostic accuracy in future clinical settings and could contribute to broader advancements in men’s health. Overall, this study emphasizes that WGS is the most effective strategy for genetic diagnosis in patients facing male fertility complications.

Our study highlights the presence of VUS and Likely Pathogenic variants even in individuals with “normal” sperm parameters. While these individuals meet the WHO criteria for normal sperm counts, motility, and morphology, the identification of specific variants in genes associated with male infertility challenges the assumption that normal sperm parameters guarantee fertility. As we have shown, males in the NG, despite their normal semen analyses, carried mutations in genes that are critical for sperm function and fertility. For example, in sample ID 275, we identified several variants in the *DNAH7* and *DNAH17* genes. Specifically, a missense SNV (*c.4457G > A; p.Arg1486His*) and another missense SNV (*c.511G > A; p.Gly171Arg*) were found in *DNAH7* on chromosome 2, and a missense SNV (*c.9404 C > T; p.Ala3135Val*) was found in *DNAH17* on chromosome 17. These variants were classified as VUS based on their CADD scores and PolyPhen2 and SIFT predictions suggest a potentially damaging effect on protein function. These findings align with previous reports showing that *DNAH7* and *DNAH17* mutations are linked to defects in sperm flagella and motility, which could affect fertility outcomes even in men with “normal” sperm parameters^53,59^.

In sample ID 280, we found two nonsense SNVs in *FSIP2*, located on chromosome 2 (*c.17425 C > T; p.Gln5809Ter* and *c.24 C > A; p.Cys8Ter*), which were predicted to introduce premature stop codons, leading to truncated proteins. These mutations were classified as Likely Pathogenic, with CADD scores, and PolyPhen2 and SIFT analyses indicating potentially damaging effects. The identification of these mutations in the NG group supports the known role of *FSIP2* in sperm flagella formation and function, and their presence in individuals with normal sperm parameters further emphasizes that genetic factors can impair fertility even in the absence of overt spermiogram abnormalities^57,59^. Additionally, in sample ID 281, we identified a missense SNV (*c.7103 C > T; p.Ser2368Phe*) in *DNAH7* and another missense SNV (*c.1672 C > T; p.Arg558Trp*) in *CATSPER1*. Both mutations were classified as VUS, with CADD scores and PolyPhen2 and SIFT predictions suggesting that they may have a damaging effect on the proteins. *CATSPER1*, a gene essential for sperm motility, has been implicated in male infertility, particularly in cases of reduced sperm motility, even when other semen parameters appear normal^30,60^. These findings suggest that mutations in *DNAH7* and *CATSPER1* could contribute to male infertility in cases where traditional sperm testing fails to detect underlying genetic causes. These results underscore the complexity of male infertility and the importance of genetic screening, even in individuals with normal semen analysis results. While sperm parameters such as count, motility, and morphology are critical factors in assessing male fertility, they do not fully capture the genetic basis of fertility problems. These results are strongly supported by previous investigations, where it was shown that semen analysis is a gold standard for evaluating male infertility. However, this technique is considered an imperfect tool^61^ because approximately 15% of men with normal semen analysis profiles have nevertheless been linked with infertility^62–64^.

Our findings align with previous research, indicating that genetic mutations in sperm-specific genes can impair fertility despite normal sperm parameters^65,66^. Human chorionic gonadotrophin (hCG) formed in the initial pregnancy can be detected in the body fluid quickly after embryo implantation. β-hCG measurement is performed in many kinds of clinical situations, like the diagnosis of pregnancy and pregnancy-related disorders. In SDIG samples, the majority have negative β-hCG results, although the IVF practice was also performed. This confirmed that negative β-hCG samples (290, 220, 271, 285, 287, and 282) have been identified with mutations in the studied genes involved in normal sperm functions. None of these patients samples (279, 201, and 203) fulfilled the variant filtering criteria that led us to the detection of a potential candidate gene responsible for male infertility. As a result, these positive β-hCG SDIG samples (279, 201, and 203) exhibited positive pregnancy. It was interesting to note that all those samples (275, 280, and 281) from the NG group where mutations were detected in sperm-specific genes (*DNAH7*, *FSIP2*, *DNAH7* & *DNAH8*, respectively) had negative β-hCG in leading to negative pregnancy. While those samples (175 and 274) with normal genomic profiles, when tested against sperm-specific genes using NGS technology, had positive β-hCG levels and ultimately resulted in a positive pregnancy.

##  Conclusion

This study highlights the complex interplay between genetic factors and male reproductive health, particularly in the context of infertility. Using whole-genome sequencing, we identified several genetic variants associated with male reproductive abnormalities, including ten variants of interest with likely pathogenic mutations in genes critical for spermatogenesis and sperm function. These findings shed light on potential diagnostic markers and underlying mechanisms, contributing to the genomic understanding of male infertility and offering a foundation for improved diagnostic approaches and the development of targeted therapeutic interventions.

Although the findings are interesting and provide valuable preliminary insights, this study has several important limitations. The relatively small sample size limits the statistical power and generalizability of our analyses, and no formal power calculation was performed prior to recruitment. Nonetheless, this work was designed as an exploratory, hypothesis-generating investigation, with a carefully selected cohort that provided meaningful initial insights despite the challenges of recruiting well-characterized SDIG cases. To reduce bias, we applied stringent filtering criteria, prioritized high-confidence variants, and cross-referenced our findings with previously reported infertility-associated genes. Notably, our analysis suggests that even individuals with normal spermiogram results may harbor genetic variants that could contribute to delayed fertilization outcomes. Another limitation is that variant classification relied primarily on the ACMG–AMP guidelines, incorporating in silico predictions, population frequency data, and prior annotations in ClinVar and the Male Fertility Gene Atlas. While this framework enabled us to distinguish likely pathogenic loss-of-function variants from missense variants of uncertain significance, the absence of in vitro functional assays or ultrastructural validation prevents definitive conclusions about pathogenicity. Furthermore, the assessment of fertility outcomes, such as IVF success, was limited to β-hCG levels, as live birth data were unavailable due to female partners delivering at another clinic.

Taken together, these results should be regarded as preliminary. Future studies incorporating larger, independent cohorts and experimental validation will be critical to confirm the biological impact of these variants, refine their clinical interpretation, and translate these insights into improved diagnostic and therapeutic strategies for male infertility.

## Supplementary Information

Below is the link to the electronic supplementary material.

Supplementary Material 1

## Data Availability

The whole genome sequencing (WGS) data in FASTQ format generated during this study have been deposited in the European Nucleotide Archive (ENA) under accession number PRJEB93831.

## References

[1] Agarwal, A., Mulgund, A., Hamada, A. & Chyatte, M. R. A unique view on male infertility around the globe. *Reprod. Biol. Endocrinol.* **13**, 37. 10.1186/s12958-015-0032-1 (2015). 25928197 10.1186/s12958-015-0032-1 PMC4424520

[2] World Health Organization. *WHO Laboratory Manual for the Examination and Processing of Human Semen*. 5th Ed. (WHO, 2010).

[3] Jungwirth, A. et al. European association of urology guidelines on male infertility: the 2012 update. *Eur. Urol.* **62**, 324–332. 10.1016/j.eururo.2012.04.048 (2012). 22591628 10.1016/j.eururo.2012.04.048

[4] Abu-Halima, M. et al. Sperm motility annotated genes: Are they associated with impaired fecundity? *Cells* **12**10.3390/cells12091239 (2023). 10.3390/cells12091239 PMC10177407 37174638

[5] Abu-Halima, M., Becker, L. S., Ayesh, B. M. & Meese, E. MicroRNA-targeting in male infertility: Sperm microRNA-19a/b-3p and its spermatogenesis related transcripts content in men with oligoasthenozoospermia. *Front. Cell. Dev. Biol.* **10**, 973849. 10.3389/fcell.2022.973849 (2022). 36211460 10.3389/fcell.2022.973849 PMC9533736

[6] Becker, L. S. et al. Towards a more comprehensive picture of the microRNA-23a/b-3p impact on impaired male fertility. *Biology (Basel)* **12**. 10.3390/biology12060800 (2023). 10.3390/biology12060800 PMC10294816 37372085

[7] Becker, L. S. et al. Proteomic landscape of human sperm in patients with different spermatogenic impairments. *Cells* **12** (2023). 10.3390/cells12071017 10.3390/cells12071017 PMC10093380 37048090

[8] Batiha, O. et al. Gene expression alterations in testicular biopsies from males with spermatogenesis arrest identified by transcriptome analysis. *PLoS One*. **20**, e0332025. 10.1371/journal.pone.0332025 (2025). 40938846 10.1371/journal.pone.0332025 PMC12431239

[9] Podgrajsek, R., Hodzic, A., Stimpfel, M., Kunej, T. & Peterlin, B. Insight into the complexity of male infertility: a multi-omics review. *Syst. Biol. Reprod. Med.* **70**, 73–90. 10.1080/19396368.2024.2317804 (2024). 38517373 10.1080/19396368.2024.2317804

[10] Wagner, A. O., Turk, A. & Kunej, T. Towards a multi-omics of male infertility. *World J. Mens Health*. **41**, 272–288. 10.5534/wjmh.220186 (2023). 36649926 10.5534/wjmh.220186 PMC10042660

[11] Jumeau, F. et al. Defining the human sperm microtubulome: an integrated genomics approach. *Biol. Reprod.* **96**, 93–106. 10.1095/biolreprod.116.143479 (2017). 28395323 10.1095/biolreprod.116.143479

[12] Xavier, M. J., Salas-Huetos, A., Oud, M. S., Aston, K. I. & Veltman, J. A. Disease gene discovery in male infertility: past, present and future. *Hum. Genet.* **140**, 7–19. 10.1007/s00439-020-02202-x (2021). 32638125 10.1007/s00439-020-02202-x PMC7864819

[13] Houston, B. J. et al. A systematic review of the validated monogenic causes of human male infertility: 2020 update and a discussion of emerging gene-disease relationships. *Hum. Reprod. Update*. **28**, 15–29. 10.1093/humupd/dmab030 (2021). 34498060 10.1093/humupd/dmab030 PMC8730311

[14] Hotaling, J. & Carrell, D. T. Clinical genetic testing for male factor infertility: current applications and future directions. *Andrology* **2**, 339–350. 10.1111/j.2047-2927.2014.00200.x (2014). 24711280 10.1111/j.2047-2927.2014.00200.x

[15] Krausz, C. & Riera-Escamilla, A. Genetics of male infertility. *Nat. Rev. Urol.* **15**, 369–384. 10.1038/s41585-018-0003-3 (2018). 29622783 10.1038/s41585-018-0003-3

[16] Cannarella, R., Condorelli, R. A., La Vignera, S. & Calogero, A. E. New perspectives in the genetic diagnosis of male infertility. *Croat Med. J.* **62**, 201–203. 10.3325/cmj.2021.62.201 (2021). 34212556 10.3325/cmj.2021.62.201 PMC8275943

[17] Zhou, H. et al. Whole exome sequencing analysis of 167 men with primary infertility. *BMC Med. Genomics*. **17**10.1186/s12920-024-02005-3 (2024). 10.1186/s12920-024-02005-3 PMC11391607 39267058

[18] Abu-Halima, M. et al. Single sperm RNA signatures reveal MicroRNA biomarkers for male subfertility. *J. Assist. Reprod. Genet.*10.1007/s10815-024-03264-w (2024). 39312032 10.1007/s10815-024-03264-w PMC11621271

[19] Li, W. N. et al. Missense mutation in DNAJB13 gene correlated with male fertility in asthenozoospermia. *Andrology* **8**, 299–306. 10.1111/andr.12685 (2020). 31342671 10.1111/andr.12685

[20] Liu, M. et al. A novel homozygous mutation in DNAJB13-a gene associated with the sperm axoneme-leads to teratozoospermia. *J. Assist. Reprod. Genet.* **39**, 757–764. 10.1007/s10815-022-02431-1 (2022). 35166991 10.1007/s10815-022-02431-1 PMC8995218

[21] El Khouri, E. et al. Mutations in DNAJB13, encoding an HSP40 family member, cause primary ciliary dyskinesia and male infertility. *Am. J. Hum. Genet.* **99**, 489–500. 10.1016/j.ajhg.2016.06.022 (2016). 27486783 10.1016/j.ajhg.2016.06.022 PMC4974111

[22] Leslie, J. S. et al. MNS1 variant associated with situs inversus and male infertility. *Eur. J. Hum. Genet.* **28**, 50–55. 10.1038/s41431-019-0489-z (2020). 31534215 10.1038/s41431-019-0489-z PMC6906318

[23] Li, Y. et al. A novel homozygous frameshift mutation in MNS1 associated with severe oligoasthenoteratozoospermia in humans. *Asian J. Androl.* **23**, 197–204. 10.4103/aja.aja_56_20 (2021). 33037173 10.4103/aja.aja_56_20 PMC7991825

[24] Hwang, J. Y. et al. Genetic defects in DNAH2 underlie male infertility with multiple morphological abnormalities of the sperm flagella in humans and mice. *Front. Cell. Dev. Biol.* **9**, 662903. 10.3389/fcell.2021.662903 (2021). 33968937 10.3389/fcell.2021.662903 PMC8103034

[25] Gao, Y. et al. Novel bi-allelic variants in DNAH2 cause severe asthenoteratozoospermia with multiple morphological abnormalities of the flagella. *Reprod. Biomed. Online*. **42**, 963–972. 10.1016/j.rbmo.2021.01.011 (2021). 33771466 10.1016/j.rbmo.2021.01.011

[26] Kumar, D., Jeena, L. M., Tempe, A., Tanwar, R. & Kumar, S. Molecular characterization of DNAH6 and ATPase6 (mitochondrial DNA) genes in asthenozoospermia patients in the Northern region of India. *BMC Urol.* **24**, 180. 10.1186/s12894-024-01505-9 (2024). 39192248 10.1186/s12894-024-01505-9 PMC11351781

[27] Whitfield, M. et al. Mutations in DNAH17, encoding a sperm-specific axonemal outer dynein arm heavy chain, cause isolated male infertility due to asthenozoospermia. *Am. J. Hum. Genet.* **105**, 198–212. 10.1016/j.ajhg.2019.04.015 (2019). 31178125 10.1016/j.ajhg.2019.04.015 PMC6612517

[28] Barbotin, A. L. et al. Identification of a novel CFAP61 homozygous splicing variant associated with multiple morphological abnormalities of the flagella. *J. Assist. Reprod. Genet.* **41**, 1499–1505. 10.1007/s10815-024-03139-0 (2024). 38775994 10.1007/s10815-024-03139-0 PMC11224159

[29] Zheng, R. et al. FSIP2 plays a role in the acrosome development during spermiogenesis. *J. Med. Genet.* **60**, 254–264. 10.1136/jmedgenet-2021-108406 (2023). 35654582 10.1136/jmedgenet-2021-108406

[30] Jalalabadi, F. N., Cheraghi, E., Janatifar, R. & Momeni, H. R. The detection of CatSper1 and CatSper3 expression in men with normozoospermia and asthenoteratozoospermia and its association with sperm parameters, fertilization rate, embryo quality. *Reprod. Sci.* **31**, 704–713. 10.1007/s43032-023-01397-4 (2024). 37957468 10.1007/s43032-023-01397-4

[31] Asenius, F. et al. The DNA methylome of human sperm is distinct from blood with little evidence for tissue-consistent obesity associations. *PLoS Genet.* **16**, e1009035. 10.1371/journal.pgen.1009035 (2020). 33048947 10.1371/journal.pgen.1009035 PMC7584170

[32] Champroux, A., Cocquet, J., Henry-Berger, J., Drevet, J. R. & Kocer, A. A decade of exploring the mammalian sperm epigenome: paternal epigenetic and transgenerational inheritance. *Front. Cell. Dev. Biol.* **6**, 50. 10.3389/fcell.2018.00050 (2018). 29868581 10.3389/fcell.2018.00050 PMC5962689

[33] Cappallo-Obermann, H. & Spiess, A. N. Comment on absence of sperm RNA elements correlates with idiopathic male infertility. *Sci. Transl Med.* **8**, 353tc351. 10.1126/scitranslmed.aaf2396 (2016). 10.1126/scitranslmed.aaf2396 27559097

[34] Martin, M. Cutadapt removes adapter sequences from high-throughput sequencing reads. *EMBnet J.* **17**, 3 (2011).

[35] Li, H. & Durbin, R. Fast and accurate short read alignment with burrows-wheeler transform. *Bioinformatics* **25**, 1754–1760. 10.1093/bioinformatics/btp324 (2009). 19451168 10.1093/bioinformatics/btp324 PMC2705234

[36] Li, H. et al. The sequence alignment/map format and samtools. *Bioinformatics* **25**, 2078–2079. 10.1093/bioinformatics/btp352 (2009). 19505943 10.1093/bioinformatics/btp352 PMC2723002

[37] Garcia-Alcalde, F. et al. Qualimap: evaluating next-generation sequencing alignment data. *Bioinformatics* **28**, 2678–2679. 10.1093/bioinformatics/bts503 (2012). 22914218 10.1093/bioinformatics/bts503

[38] McKenna, A. et al. The genome analysis toolkit: a mapreduce framework for analyzing next-generation DNA sequencing data. *Genome Res.* **20**, 1297–1303. 10.1101/gr.107524.110 (2010). 20644199 10.1101/gr.107524.110 PMC2928508

[39] Cingolani, P. et al. A program for annotating and predicting the effects of single nucleotide polymorphisms, snpeff: SNPs in the genome of drosophila melanogaster strain w1118; iso-2; iso-3. *Fly. (Austin)*. **6**, 80–92. 10.4161/fly.19695 (2012). 22728672 10.4161/fly.19695 PMC3679285

[40] Kopanos, C. et al. VarSome: the human genomic variant search engine. *Bioinformatics* **35**, 1978–1980. 10.1093/bioinformatics/bty897 (2019). 30376034 10.1093/bioinformatics/bty897 PMC6546127

[41] Rentzsch, P., Witten, D., Cooper, G. M., Shendure, J. & Kircher, M. CADD: predicting the deleteriousness of variants throughout the human genome. *Nucleic Acids Res.* **47**, D886–D894. 10.1093/nar/gky1016 (2019). 30371827 10.1093/nar/gky1016 PMC6323892

[42] Uhlen, M. et al. Proteomics. Tissue-based map of the human proteome. *Science* **347**, 1260419. 10.1126/science.1260419 (2015). 25613900 10.1126/science.1260419

[43] Richards, S. et al. Standards and guidelines for the interpretation of sequence variants: a joint consensus recommendation of the American College of Medical Genetics and Genomics and The Association for Molecular Pathology. *Genet. Med.* **17**, 405–424. 10.1038/gim.2015.30 (2015). 25741868 10.1038/gim.2015.30 PMC4544753

[44] Bhattacharya, I., Sharma, S. S. & Majumdar, S. S. Etiology of male infertility: an update. *Reproductive Sci.* **31**, 942–965. 10.1007/s43032-023-01401-x (2024). 10.1007/s43032-023-01401-x 38036863

[45] Inaba, K. & Mizuno, K. Sperm dysfunction and ciliopathy. *Reprod. Med. Biol.* **15**, 77–94. 10.1007/s12522-015-0225-5 (2016). 29259424 10.1007/s12522-015-0225-5 PMC5715842

[46] Li, Y. et al. DNAH2 is a novel candidate gene associated with multiple morphological abnormalities of the sperm flagella. *Clin. Genet.* **95**, 590–600. 10.1111/cge.13525 (2019). 30811583 10.1111/cge.13525

[47] Guan, J., Ekwurtzel, E., Kvist, U., Hultenby, K. & Yuan, L. DNAJB13 is a radial spoke protein of mouse ‘9 + 2’ axoneme. *Reprod. Domest. Anim.* **45**, 992–996. 10.1111/j.1439-0531.2009.01473.x (2010). 19919626 10.1111/j.1439-0531.2009.01473.x

[48] Ware, S. M., Aygun, M. G. & Hildebrandt, F. Spectrum of clinical diseases caused by disorders of primary cilia. *Proc. Am. Thorac. Soc.* **8**, 444–450. 10.1513/pats.201103-025SD (2011). 21926397 10.1513/pats.201103-025SD PMC3209578

[49] Ta-Shma, A. et al. Homozygous loss-of-function mutations in MNS1 cause laterality defects and likely male infertility. *PLoS Genet.* **14**, e1007602. 10.1371/journal.pgen.1007602 (2018). 30148830 10.1371/journal.pgen.1007602 PMC6128653

[50] Li, L. et al. DNAH6 is a novel candidate gene associated with sperm head anomaly. *Andrologia*10.1111/and.12953 (2018). 29356036 10.1111/and.12953

[51] Kao, S. H., Chao, H. T., Liu, H. W., Liao, T. L. & Wei, Y. H. Sperm mitochondrial DNA depletion in men with asthenospermia. *Fertil. Steril.* **82**, 66–73. 10.1016/j.fertnstert.2003.11.056 (2004). 15236991 10.1016/j.fertnstert.2003.11.056

[52] Kao, S. H., Chao, H. T. & Wei, Y. H. Multiple deletions of mitochondrial DNA are associated with the decline of motility and fertility of human spermatozoa. *Mol. Hum. Reprod.* **4**, 657–666. 10.1093/molehr/4.7.657 (1998). 9701788 10.1093/molehr/4.7.657

[53] Gao, Y. et al. Loss of function mutation in DNAH7 induces male infertility associated with abnormalities of the sperm flagella and mitochondria in human. *Clin. Genet.* **102**, 130–135. 10.1111/cge.14146 (2022). 35543642 10.1111/cge.14146

[54] Zhou, Z. et al. A novel splicing variant in DNAH8 causes asthenozoospermia. *J. Assist. Reprod. Genet.* **38**, 1545–1550. 10.1007/s10815-021-02116-1 (2021). 33611675 10.1007/s10815-021-02116-1 PMC8266938

[55] Tang, X. et al. Novel variants in DNAH17 cause sperm flagellar outer dynein arm defects but not total fertilization failure after ICSI. *Reprod. Biomed. Online*. **50**, 104492. 10.1016/j.rbmo.2024.104492 (2024). 40009974 10.1016/j.rbmo.2024.104492

[56] Knowles, M. R., Zariwala, M. & Leigh, M. Primary ciliary dyskinesia. *Clin. Chest. Med.* **37**, 449–461 (2016). 27514592 10.1016/j.ccm.2016.04.008 PMC4988337

[57] Liu, M. et al. Novel mutations in FSIP2 lead to multiple morphological abnormalities of the sperm flagella and poor ICSI prognosis. *Gene* **781**, 145536. 10.1016/j.gene.2021.145536 (2021). 33631238 10.1016/j.gene.2021.145536

[58] Bracke, A., Peeters, K., Punjabi, U., Hoogewijs, D. & Dewilde, S. A search for molecular mechanisms underlying male idiopathic infertility. *Reprod. Biomed. Online*. **36**, 327–339 (2018). 29336995 10.1016/j.rbmo.2017.12.005

[59] Arora, M. et al. Genetic etiological spectrum of sperm morphological abnormalities. *J. Assist. Reprod. Genet.* **41**, 2877–2929. 10.1007/s10815-024-03274-8 (2024). 39417902 10.1007/s10815-024-03274-8 PMC11621285

[60] Hildebrand, M. S. et al. Genetic male infertility and mutation of CATSPER ion channels. *Eur. J. Hum. Genet.* **18**, 1178–1184. 10.1038/ejhg.2010.108 (2010). 20648059 10.1038/ejhg.2010.108 PMC2987470

[61] Patrizio, P., Sanguineti, F. & Sakkas, D. Modern andrology: from semen analysis to postgenomic studies of the male gametes. *Ann. N Y Acad. Sci.* **1127**, 59–63. 10.1196/annals.1434.021 (2008). 18443330 10.1196/annals.1434.021

[62] Agarwal, A. & Allamaneni, S. S. Sperm DNA damage assessment: a test whose time has come. *Fertil. Steril.* **84**, 850–853 (2005). 16213833 10.1016/j.fertnstert.2005.03.080

[63] Spano, M., Seli, E., Bizzaro, D., Manicardi, G. C. & Sakkas, D. The significance of sperm nuclear DNA strand breaks on reproductive outcome. *Curr. Opin. Obstet. Gynecol.* **17**, 255–260. 10.1097/01.gco.0000169102.77504.66 (2005). 15870559 10.1097/01.gco.0000169102.77504.66

[64] Lin, M. H. et al. Sperm chromatin structure assay parameters are not related to fertilization rates, embryo quality, and pregnancy rates in in vitro fertilization and intracytoplasmic sperm injection, but might be related to spontaneous abortion rates. *Fertil. Steril.* **90**, 352–359. 10.1016/j.fertnstert.2007.06.018 (2008). 17904130 10.1016/j.fertnstert.2007.06.018

[65] Avenarius, M. R. et al. Human male infertility caused by mutations in the CATSPER1 channel protein. *Am. J. Hum. Genet.* **84**, 505–510. 10.1016/j.ajhg.2009.03.004 (2009). 19344877 10.1016/j.ajhg.2009.03.004 PMC2667975

[66] Zhou, J. et al. A recessive ACTL7A founder variant leads to male infertility due to acrosome detachment in Pakistani Pashtuns. *Clin. Genet.* **104**, 564–570. 10.1111/cge.14383 (2023). 37286336 10.1111/cge.14383

