# Supplementary material for: Identifying potential genetic biomarkers for sperm dysfunction through whole-genome sequencing
Source: Sci Rep. 2025 Oct 20;15:36476. doi: 10.1038/s41598-025-23897-w (PMC12537905; doi:10.1038/s41598-025-23897-w)
Supplement: Supplementary file 1 — Supplementary Material 1 [file 41598_2025_23897_MOESM1_ESM.pdf]

Identifying Potential Genetic Biomarkers for Sperm Dysfunction through Whole-Genome Sequencing

Muhammad Riaz Khan, Ph.D., [1], Aftab Ali Shah, Prof. Ph.D., [1], Mohammad A. Al Smadi, Ph.D., [2], Nicole Ludwig, Ph.D., [3], Ulrike Fischer, Prof. Dr., [3], Hashim Abdul-Khaliq, Prof. Dr., [4], Eckart Meese, Prof. Dr., [3], Masood Abu-Halima, Ph.D., [3, 4]

Affiliations

[1] Department of Biotechnology, Faculty of Biological Sciences, University of Malakand, Chakdara, Khyber Pakhtunkhwa, Pakistan.

[2] Reproductive Endocrinology and IVF Unit, King Hussein Medical Centre, Amman, Jordan.

[3] Institute of Human Genetics, Saarland University, Homburg, Germany.

[4] Department of Paediatric Cardiology, Saarland University Hospital, Homburg, Germany.

Corresponding Author

Masood Abu-Halima
Saarland University
Institute of Human Genetics
66421 Homburg, Germany
Tel: +49 (0) 6841 16 26289
Fax: +49 (0) 6841 16 26185
Email: masood@daad-alumni.de

Supplementary Tables

Supplemental Table S1| PCR Primer Sequences and Parameters for Amplification of Selected Genes

Sample ID	Gene	NT Genomic Change (GRCh38)	Forward Primer	Tm	Reverse Primer	Tm	Product Size
220	MNS1	chr15:56444481 C>T	GTCCTCTGACATATGATATGGGCA	59.8	AACAGAAACGTGATGCTGAAAT	57.2	508 bp

285	DNAH6	chr2:84677021 C>T	TTAACCATCCAGGGCCTTGG	59.7	AATGCCTGCCAGCTACTCTC	59.8	505 bp
287	CFAP61	chr20:20196681 C>T	TACAAGCTGTGCTGCCATCC	60.7	CGGCGTACAACACCAACAAC	60.3	505 bp

Supplemental Table S2| Comprehensive comparison of genomic metrics between NG and SDIG.

**Sample ID**	**Mapped reads** (count)	**Reads Mapped (%)**	**GC (%)**	**Coverage (x)**	**Mapping Quality**
290	333606207	99.51	40.01	14.81	31.66
220	257935167	99.83	40.12	11.68	31.94
279	435981099	99.82	39.95	19.70	31.95
271	439189139	99.23	39.92	19.86	32.03
201	308445778	99.87	39.16	13.95	31.93
285	285367834	99.78	39.46	12.58	31.99
203	277992846	99.83	39.13	12.56	32.06
287	504887326	96.72	39.84	22.62	31.90
282	341593902	99.72	39.89	15.05	31.92
**Average (SDIG)**	**359697527**	**99.45**	**39.63**	**15.90**	**31.94**

**Sample ID**	**Mapped reads (count)**	**Reads Mapped %**	**GC%**	**Coverage (x)**	**Mapping Quality**
270	357309010	99.89	39.08	15.76	31.99
175	231027751	99.83	39.52	10.44	31.81
251	428168909	99.88	39.20	19.07	31.99
280	384585238	99.89	39.64	16.91	31.97
275	372978500	99.88	39.67	16.89	32.07
286	366062055	99.87	39.68	16.15	31.79
281	348975229	99.85	40.02	15.38	31.83
274	294999670	99.85	40.13	13.30	31.97
**Average (NG)**	**349687682**	**99.87**	**39.59**	**15.47**	**31.92**

Supplemental Table S3| Analysis of Genetic Consequences in NIG and SDIG.

**Consequence**	**SDIG (n=9)**	**NG (n=8)**
**Transcript ablation**	7	8
Splice acceptor variant	668	629
Splice donor variant	873	792
Stop gained	640	551
Frameshift variant	1380	1220
Stop lost	183	151
Start lost	75	72
Inframe insertion	834	700
Inframe deletion	1041	895

Missense variant	65847	58685
Splice donor 5th base variant	988	872
Splice region variant	20421	18052
Splice donor region variant	3258	2938
Splice polypyrimidine tract variant	29851	26231
Incomplete terminal codon variant	5	7
Protein altering variant	19	8
Start retained variant	22	10
Stop retained variant	5961	72
Synonymous variant	64687	62735
Coding sequence variant	46	36
Mature miRNA variant	79	62
5' UTR variant	24957	54829
3' UTR variant	294022	226957
Non-coding transcript exon variant	237966	210395
Intron variant	18240292	16151864
NMD transcript variant	71854	63234
Non-coding transcript variant	5753263	5127880
Upstream gene variant	1956443	1711432
Downstream gene variant	1859125	1627935
TFBS ablation	357	272
TF binding site variant	100545	88707
Regulatory region variant	1137540	1014734
Intergenic variant	11088515	9809232

Supplemental Figure S1| The expression profiles of the candidate were analyzed. For each gene, the expression level is shown in two ways: on the left, across 54 body tissues based on bulk RNA sequencing data from the GTEx project; on the right, at single-cell resolution within cells of the adult testis, as summarized by the Human Infertility Single-Cell Testis Atlas (HISTA).

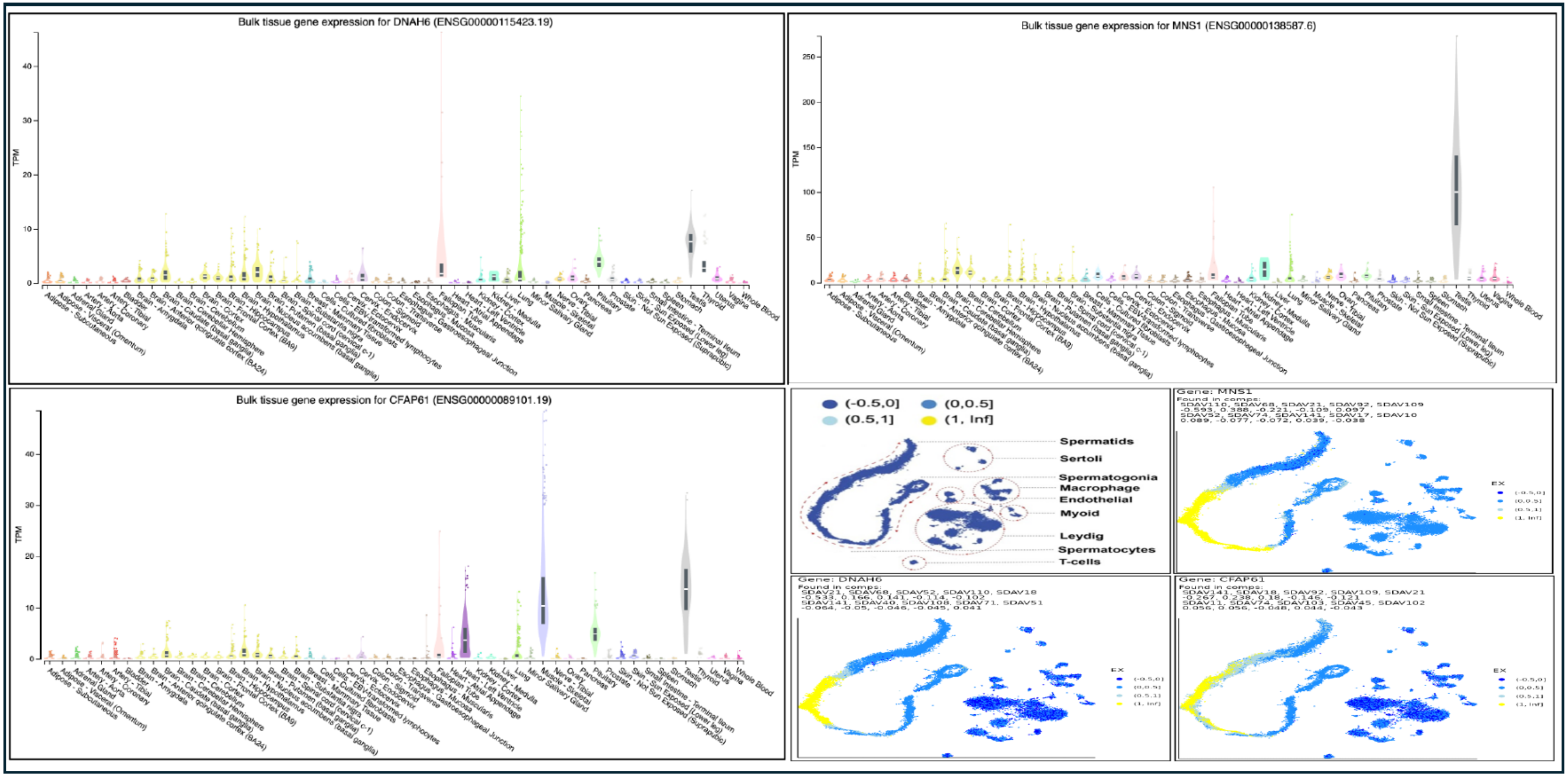
Bulk tissue gene expression for DNAH6 (ENSG00000115423.19)
Bulk tissue gene expression for MNS1 (ENSG00000138587.6)
Bulk tissue gene expression for CFAP61 (ENSG00000089101.19)
TPM
(-0.5,0]
(0,0.5]
(0.5,1]
(1, Inf]
Spermatids
Sertoli
Spermatogonia
Macrophage
Endothelial
Myoid
Leydig
Spermatocytes
T-cells
Gene: MNS1
Found in comps:
SDAV110, SDAV68, SDAV21, SDAV92, SDAV109
-0.593, 0.388, -0.221, -0.109, 0.097
SDAV52, SDAV74, SDAV141, SDAV17, SDAV10
0.089, -0.077, -0.072, 0.039, -0.038
Gene: DNAH6
Found in comps:
SDAV21, SDAV68, SDAV52, SDAV110, SDAV18
-0.533, 0.166, 0.141, -0.114, -0.102
SDAV141, SDAV40, SDAV108, SDAV71, SDAV51
-0.064, -0.05, -0.046, -0.045, 0.041
Gene: CFAP61
Found in comps:
SDAV141, SDAV18, SDAV92, SDAV109, SDAV21
-0.267, 0.238, 0.18, -0.146, -0.121
SDAV11, SDAV74, SDAV103, SDAV45, SDAV102
0.056, 0.056, -0.048, 0.044, -0.043
EX
